# Dr. R. S. Lavenson

**Published:** 1913-09

**Authors:** 


Dr. R. S. Lavenson, formerly of Philadelphia, died in Los Angeles July 6 from tuberculosis, acquired during research work.

